# Effect of protein source and resistance training on body composition and sex hormones

**DOI:** 10.1186/1550-2783-4-4

**Published:** 2007-07-23

**Authors:** Douglas Kalman, Samantha Feldman, Michele Martinez, Diane R Krieger, Mark J Tallon

**Affiliations:** 1 Miami Research Associates, Nutrition/Endocrinology Division, Miami, Florida, USA

## Abstract

**Background:**

Evidence suggests an inverse relationship between soy protein intake and serum concentrations of male sex hormones. Anecdotal evidence indicates that these alterations in serum sex hormones may attenuate changes in lean body mass following resistance training. However, little empirical data exists regarding the effects of soy and milk-based proteins on circulating androgens and exercise induced body composition changes.

**Methods:**

For 12 weeks 20 subjects were supplemented with 50 g per day of one of four different protein sources (Soy concentrate; Soy isolate; Soy isolate and whey blend, and Whey blend only) in combination with a resistance-training program. Body composition, testosterone, estradiol and sex hormone binding globulin (SHBG) were measured at baseline and week 12.

**Results:**

Protein supplementation resulted in a significant increase in lean body mass independent of protein source (0.5 ± 1.1 and 0.9 ± 1.4 kg, *p *= 0.006, *p *= 0.007). No significant differences were observed between groups for total and free testosterone, SHBG, percentage body fat, BMI or body weight. The Testosterone/Estradiol ratio increased across all groups (+13.4, *p *= 0.005) and estradiol decreased (*p *= 0.002). Within group analysis showed significant increases in the Testosterone/Estradiol ratio in soy isolate + whey blend group (+16.3, *p *= 0.030). Estradiol was significantly lower in the whey blend group (-9.1 ± 8.7 pg/ml, *p *= 0.033).

**Conclusion:**

This investigation shows that 12 week supplementation with soy protein does not decrease serum testosterone or inhibit lean body mass changes in subjects engaged in a resistance exercise program.

## Background

The maintenance of skeletal muscle mass can be defined as the net result of protein synthesis and degradation. In most healthy persons the consumption of regular meals without training results in a relatively stable balance of muscle tissue over time. Generally it is acknowledged that a combination of training and adequate nutrition promotes the accretion of lean body tissue; the presence of a training stimulus and a positive protein balance fosters skeletal muscle fiber hypertrophy [1,2].

An aspect of adequate nutritional intake is related to the bioavailability of ingested dietary protein and its source (i.e. essential amino acids) which can directly influence the magnitude of adaptation of a given training stimulus. Previous work has suggested that soy protein is preferentially directed towards the splanchnic region and milk proteins, (i.e. whey and casein), to peripheral regions such as muscle tissues [3,4]. When compared to soy proteins, milk proteins provide greater amounts of the branched chain amino acids (BCAAs) leucine, isoleucine and valine, as well as methionine and lysine [5-8]. Recent work has identified the importance of the BCAA leucine in the activation of myogenic translation initiation factors such as MTOR, P70 S6 kinase and eIF4E – eIF4G [for review see: [9-11]], which are considered important for muscle hypertrophy. As such the relatively low BCAA content found in soy protein may decrease the effectiveness of downstream leucine signaling [5].

Phytoestrogens (isoflavones) are a group of natural estrogen receptor modulators that are highly concentrated in soy foods, including soy protein isolates [12]. Soy isoflavones have comparable molecular weights and are structurally similar to 17-beta estradiol, which may enable them to exert estrogenic and antiestrogenic activities through their associated receptor-binding site [13]. In-vitro studies lend support to this relationship by demonstrating the ability of soy to inhibit a variety of androgenic and estrogenic hormones including: testosterone [14], sex hormone binding globulin (SHBG) [15], esterone [16] and testosterone/estradiol ratio [17]. However, in-vivo evidence shows that the source and concentration of soy isoflavones does not impact levels of circulating sex hormones [18-20]. It has been demonstrated circulating sex hormone levels are closely linked to the adaptive response to resistance exercise [21]. This provides one such premise regarding the perception that soy protein sources are inferior to milk proteins such as whey for supporting lean mass accretion in males engaged in resistance exercise [22].

Although there is some evidence regarding the benefits of whey over soy as an efficient adaptogenic protein source for muscle tissue, no human studies have compared the two proteins directly in response to resistance training. Based on this background, the present study assessed the effect of 12 weeks of resistance training and dietary supplementation with soy, whey or a combination, on body composition and plasma sex hormone concentrations.

## Methods

### Subjects and study design

Forty-one potential subjects were recruited from the local community and screened using a brief phone call and questionnaire. If the initial screening led to selection as a potential candidate for inclusion, renal and liver function tests were performed. Inclusion criteria included healthy males 18 – 40 years of age with a BMI of < 40 kg/m^2^. Exclusion criteria included the following: (*a*) Self professed vegetarians, or followers of ketogenic or carbohydrate restrictive diets, (*b*) Supplemental creatine users, (*c*) Subjects with renal and liver function test higher than 1.5 – 2 × normal, (*d*) Subjects that had used anabolic steroids within the last year and/or were using stimulants over the past 90 days, (*e*) Subjects who had experienced weight loss or gain greater than 4.5 kg over the previous 90 days. Twenty young healthy men (age, 30.7 ± 6.5 years; height, 176.3 ± 7.7 cm; weight, 82.0 ± 12.3 kg; mean ± SD) completed the 12 week study. All aspects of the investigation were first approved by the Ethics Review Committee of *IntegReview *(Austin, TX). Subjects were informed of experimental procedures and signed informed consent statements according to human subject guidelines of the Declaration of Helsinki, The US-FDA guidelines and those of *IntegReview*. The subjects were matched according to training experience, experienced (greater than 3 months) and inexperienced (less than 3 months), and randomly assigned to either soy isolate, soy concentrate, whey blend, or soy isolate plus whey blend group. Subject characteristics are outlined in Table 1. To maintain study and subject blinding, neither researchers nor subjects knew which group they belonged to until completion of the trial and laboratory analysis. Safety (clinical blood analysis, adverse event [AE] records) and efficacy end points (body weight and composition change) were assessed at baseline and end-of-study (week 12).

**Table 1:** Subject characteristics at pre-training

	Group 1 (SI) *n = 5*	Group 2 (SC) *n = 5*	Group 3 (WB) *n = 5*	Group 4 (SW) *n = 5*
Age (y)	30.3 ± 8.1	31.6 ± 5.9	31.4 ± 5.1	29.6 ± 7.0
Weight (kg)	82.1 ± 20.3	86.1 ± 6.9	80.9 ± 8.7	78.7 ± 13.3
Height (cm)	175.8 ± 13.7	172.1 ± 8.5	177.6 ± 5.2	179.8 ± 3.5
BMI (kg/m^2^)	28.2 ± 6.1	29.2 ± 3.7	26.4 ± 3.0	24.4 ± 4.6
Test_tot _(ng/dl)	522 ± 69.4	649.6 ± 205.3	609.3 ± 164.9	522.4 ± 188.1
Test_free _(pg/ml)	101.1 ± 11.9	146.7 ± 38.4	128.2 ± 47.7	101.4 ± 21.4
Estradiol (pg/ml)	21.7 ± 6.8	24.2 ± 2.3	27.1 ± 8.6	20.0 ± 4.1
SHBG (nM/l)	24.3 ± 11.2	23.2 ± 7.1	21.3 ± 21.1	29.4 ± 14.2
Test_free_/Estradiol ratio	25.3 ± 5.7	26.9 ± 8.2	25.0 ± 10.7	26.5 ± 9.7

Data are mean ± SD. No significant pre-training group differences were found (*p *< 0.05) n = 20

### Supplementation

Subjects had diets supplemented with four study protein powders: soy protein isolate (SI), soy concentrate (SC), whey blend (50% whey concentrate, 50% whey isolate) (WB) or a 50:50 mixture of soy isolate and whey blend (SW). Study treatment powders were analyzed in duplicate by HPLC for nutritional composition including isoflavone content (Nestle Purina Analytical Labs, St Louis, USA). Soy protein was supplied by the Solae Company, LLC and whey proteins from Land O'Lakes, Inc. Batch sample analyses are outlined in Table 2. On training days, subjects were instructed to dissolve one serving (25 g) of protein supplement in 10–12 oz of water and ingest within 1 h following training; a second dose was consumed later in the day. On non-training days subjects consumed two doses of protein at different times throughout the day. Product compliance was assessed upon weekly returns of empty packets to the laboratory. Packaging and taste were masked to disguise identification of the protein supplements.

**Table 2:** Composition of supplements used in experimental interventions

**Active Compound**	**SI**	**SC**	**SW**	**WB**
Protein (%)	59	59.5	59.5	60
Calcium^#^	304	418	379	438
Ash (%)	5.1	6.7	5.3	5.5
Fat, acid hydrolysis (%)	2.3	2.6	1.8	2.7
Moisture (%)	3.6	3.2	3.5	3.6
Phosphorus^#^	626	813	481	295
Potassium^#^	1450	1540	1530	1410
Sodium^#^	491	539	536	574
Genistein-containing compounds^#^	62	49	30	10
Daidzein-containing compounds^#^	29	107	14	2
Glycitein-scontaining compounds^#^	7	20	4.0	0
Genistein^#^	37	83	18	1.0
Daidzein^#^	17	59	8	1.0
Glycitein^#^	4	12	2.0	0
Total aglycone Components^#^	58	154	29	2
Total isoflavones^#^	98	276	48	3

Mean data values

^# ^Concentration = mg/100 g

### Training and mood profiling

Weight training was performed 3 days per week for 12 weeks with individual instruction once per week by a qualified personal trainer to ensure maintenance of adequate intensity, training load, and correct form were followed as per protocol. Program design was in accordance with and based on the American College of Sports Medicine recommendations for hypertrophy [23]. The hypertrophic training protocol consisted of 3–4 sets of 8–12 repetitions per set and 1–2 minutes rest between sets. The following exercises were performed to emphasize multi-joint exercise for large then smaller muscle groups, respectively: 1) Bench press; 2) Military press; 3) Bicep curls; 4) Tricep pushdowns 5) Leg press or Squat; 6) Supine leg curls; 7) Leg extensions; 8) Calf rises and 9) Abdominal crunches. It was recommend that subjects rest for 48 h between training the same body-part to allow for adequate recovery. Profile of mood states for fatigue and vigor (POMS-F/V) [15] were recorded at baseline and week 12.

### Biochemical body composition analyses

Following an overnight fast blood samples were obtained pre study (screen) and weeks 0 (baseline), and 12 for each intervention group. Blood samples were also collected at week 6 to assess patients metabolic panel including CBC w/differential as part of study safety protocol. Subjects were requested to refrain from food and beverages (except water) for 12 h prior to each blood draw. Blood was drawn from the antecubital vein into anti-coagulant free tubes centrifuged at 1200 × g at 4°C and serum removed within 5 min of separation. Serum aliquots were then divided and stored at -80°C until analysis. Serum samples underwent duplicate analysis for complete the metabolic panel and CBC w/differential, androgens, estrogens, and SHBG (Quest Diagnostics, Miramar, Florida). Serum total and free testosterone were analyzed using Liquid chromatography mass spectrometry (LC/MS) at a commercial laboratory (Quest Diagnostics, Miramar, FL.). SHBG and estradiol were analyzed using an immunoradiometric assay with I-labeled antibodies. Intra- and inter-assay variability was 8.3% and 8.6% for Test_free_, 9.4% and 11.7% for Test_Tot_, 7.2% and 9.46% for estradiol, respectively. Body composition was determined  at weeks 0 and 12 by Dual-Energy X-Ray Absorptiometry (DEXA; Hologic QDR  4500W v 11.2).

### Food recording

Each subject received detailed instruction before completing a multiple pass 24 h dietary recall at baseline and post interventions [24]. Subjects were encouraged to provide as much detail as possible including submission of food labels and recipes. Food records were analyzed using Nutritionist Pro™ Ver. 1.1 (Hearst Corporation, CA) and for each 24 h dietary recall, mean and SD intakes of energy and macronutrients were calculated [24].

### Statistical analysis

Treatment effects were assessed by comparing the amount of change from baseline to end-of-study among the four formulation groups by a one-way, four-level factorial analysis of variance (ANOVA) with Tukey HSD post-hoc tests. Changes over time from baseline to each subsequent visit within each formulation group were assessed for significance by the one-sample Students *t*-test (testing whether the change from baseline was significantly different from zero). Analysis (per protocol, finishers only) were undertaken on only 20 of the initial 41 subjects were full data collection, product compliance greater than 50%, and all laboratory and training sessions attended. Where appropriate, Fisher's Exact test was utilized for comparison of event rates. The sample size for this pilot study was predetermined, and not obtained by a formal power calculation. Thus no formal pre-study power analysis was undertaken and the data is considered inferential and not conclusive. Statistical analyses (descriptive statistics and Students *t*-tests) were performed using SPSS ver.11.5 (SPSS Inc., Chicago IL). The Fisher's Exact test, and the analysis of the alpha and power characteristics of the hybrid study design, were performed using R ver.1.9.1. An alpha level of *p *< 0.05 was considered significant.

## Results

### Subject characteristics

**Figure 1:**
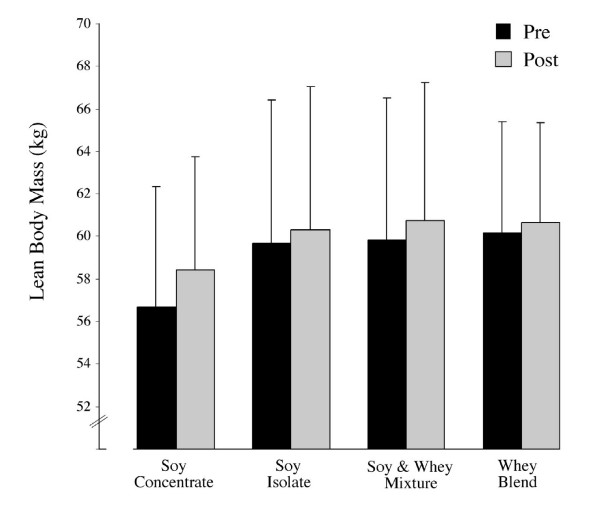
Mean (± SD) lean body mass measured at baseline and following 12 weeks of protein supplementation and resistance exercise. At week 12, group analysis shows a significant enhancement in lean mass (0.9 kg, *p *= 0.007). A trend towards significance within groups was present only in the soy isolate group (*p *= 0.055).

Subject (*n *= 20) characteristics taken at baseline are given in Table 1. A significant increase in all groups in lean body mass was present (0.9 kg, *p *= 0.007) however following a within group analysis a trend towards significance for an increase in lean body mass was present only in the SI group (*p *= 0.055) (Figure 1). During the study, BMI and body weight increased but not significantly and percentage body fat did not change. No important adverse events occurred that were likely related to the use of the protein supplements and as such no significant AE rates were reported across the four formulation groups. Some isolated changes in metabolic panel and CBC w/differential were noted although not clinically important as defined within the normal clinical range, or consistently occurring across supplemental groups when assessed at baseline, midpoint or week 12.

### Dietary intakes

The intakes of energy and macronutrients based on 24 h dietary recall showed significant differences among the formulations for kcals and fat, respectively (-555 ± 1029; *p *= 0.026; -27 ± 41 g/day, *p *= 0.009). A comparison of food records over the 12-week study period revealed that subjects consumed significantly more protein (+63 g/day, *p *= 0.013) in the SC group at baseline compared to week 12 (Table 3).

**Table 3:** Changes in baseline versus end study dietary analysis following PPA

	**SC**	**SI**	**SW**	**WB**	Total	p
**Caloric Intake **Kcals/day	141 ± 386 p = 0.461	-465 ± 494 p = 0.244	-1558 ± 1298 p = 0.055	-373 ± 875 p = 0.302	-555 ± 1029 p = 0.026	0.047
**Protein **gms/day	63 ± 33 p = 0.013	-20 ± 38 p = 0.244	-21 ± 38 p = 0.276	23 ± 109 p = 0.596	16 ± 75 p = 0.364	0.275
**Carbohydrates **Gms/day	27 ± 104 p = 0.588	-41 ± 49 p = 0.278	-246 ± 314 p = 0.155	-82 ± 104 p = 0.084	-89 ± 193 p = 0.052	0.143
**Fats **Gms/d	-24 ± 29 p = 0.143	-22 ± 16 p = 0.146	-51 ± 68 p = 0.168	-13 ± 28 p = 0.267	-27 ± 41 p = 0.009	0.481

Data are mean ± SD. Significant changes are indicated using an alpha level of p < 0.05.

### Serum sex hormones and SHBG

**Figure 2:**
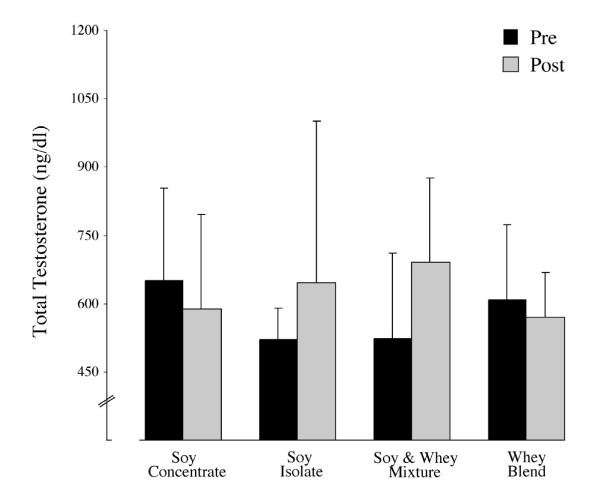
Mean (± SD) of total testosterone at baseline and following 12 weeks of protein supplementation and resistance exercise. No significant changes between or within groups were evident at week 12.

**Figure 3:**
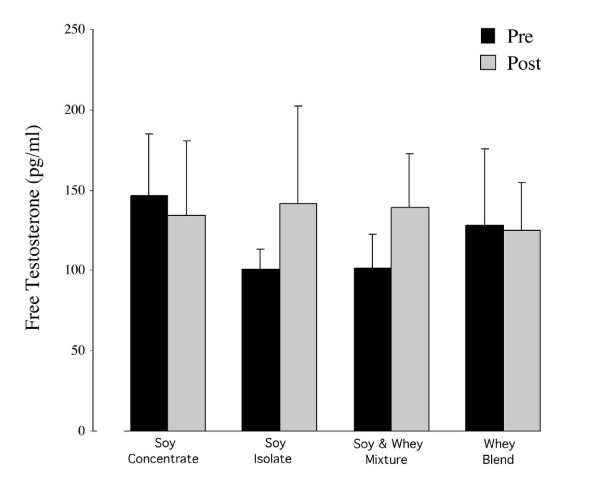
Mean (± SD) of free testosterone at baseline and following 12 weeks of protein supplementation and resistance exercise. No significant changes between groups were evident at week 12.

**Figure 4:**
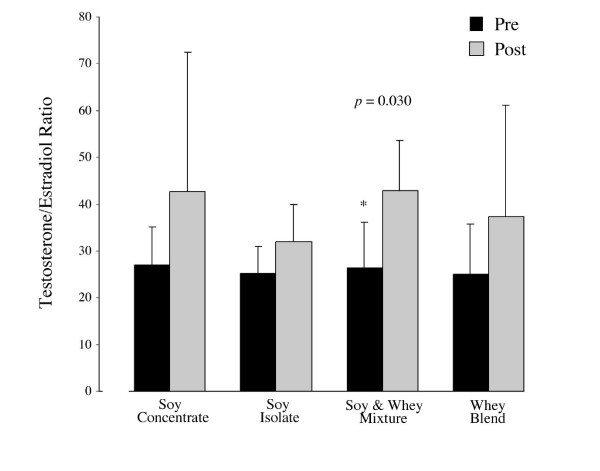
Mean (± SD) of testosterone/estradiol ratio following 12 weeks of protein supplementation and resistance exercise. As significant increase in the testosterone/estradiol ratio between groups was noted (+13.4, p = 0.005). The testosterone/estradiol ratio within the SW group was higher at week 12 in comparison to baseline measurements (+16.3, p = 0.030). * Indicates significant difference (p < 0.05)

**Figure 5:**
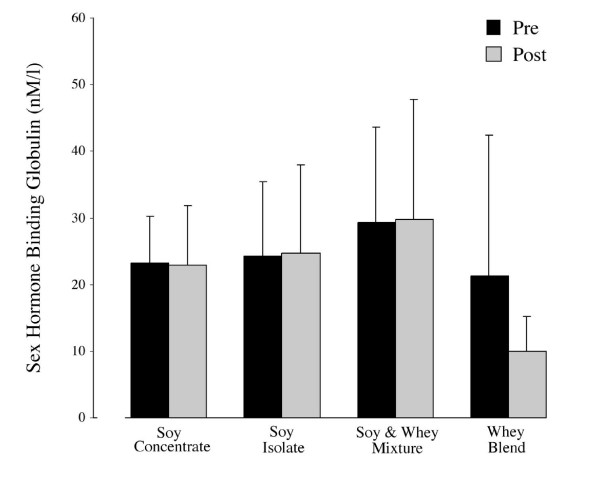
Mean (± SD) of SHBG at baseline and following 12 weeks of protein supplementation and resistance exercise. No significant changes between or across groups were evident at week 12.

Over the study period serum measurements of free and total testosterone did not differ significantly between or within groups (Figures 2 and 3). However, a significant increase was noted in the testosterone/estradiol ratio across groups (+13.4, *p *= 0.005). Specifically, the testosterone/estradiol ratio in the SW group was higher at week 12 in comparison to baseline measurements (+16.3, *p *= 0.030) (Figure 4). The effects of supplementation on SHBG within and across all groups were not statistically significant (Figure 5).

**Figure 6:**
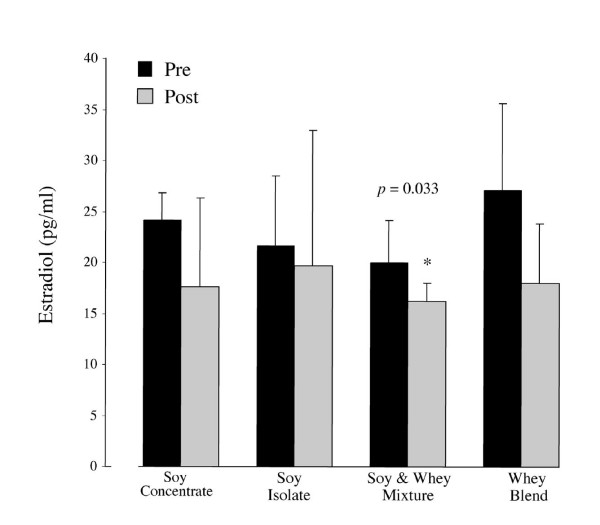
Mean (± SD) of estradiol at baseline and following 12 weeks of protein supplementation and resistance exercise. Serum estradiol was significantly lower in all four groups following supplementation and training (p = 0.002). Within group analysis shows a significant decrease in the WB intervention was statistically significant (-9.1 pg/ml, p = 0.033). * Indicates significant difference (p < 0.05)

Group serum estradiol was significantly lowers following supplementation and training (*p *= 0.002). A significant within group decrease in the WB intervention was evident (-9.1 pg/ml, *p *= 0.033) and although not significant a trend towards lower values was observed in all other groups (Figure 6).

### Profile of mood states (POMS)

No significant change over the course of the supplementation period was observed for POMS fatigue within and across groups. The effects of protein supplementation and training on POMS vigor over the 12 week period did not significantly change across groups. However, a significant decrease in POMS vigor was noted in the SC group (+3.2, *p *= 0.040).

## Discussion

This study is the first to investigate the effects of whey and soy protein supplements on androgenic hormones during 12 weeks of resistance training. The main finding was that 12 weeks of resistance training induced similar gains in lean body mass in subjects consuming a diet supplemented with whey or soy protein (Figure 1). This finding is contrary to the hypothesis that soy is an inferior source of protein in comparison to whey.

The SI supplemented group neared significance regarding lean mass increases over the 12 week training schedule (Figure 1). Previously, studies over a similar time frame have shown no significant differences between soy and whey supplementation with equal protein content [25]. This suggests that over the short term the bioactive composition of these proteins has little additive effect on the magnitude of change in lean tissue mass. However, the subjects enrolled in the study were relatively novice exercisers with little previous exposure to training program. This may result in only early stage hypertrophic adaptation [26]. The rationale is that early adaptive response to resistance exercise is neuromuscular in etiology and subsequent skeletal muscle fiber hypertrophy plays only a minor part in the total adaptive response [26].

Soy is well known for its estrogenic effects, which may occur through the hypothalamic-pituitary-gonadal axis [27]. Isoflavones inhibit key steroidogenic linked enzymes including aromatase enzyme, 17β-hydroxysteroid oxidoreductase and cytochrome p-450 with the latter responsible for estrogen hydroxylation [28,27,4]. These postulated mechanisms provide a probable rationale for the decline in serum estradiol across all soy groups within the current study (Figure 6). Reduction in serum estrogens has previously been shown following soy-milk supplementation for 8 weeks [29]. A declining trend in estradiol was also evident following supplementation in the WB group, although not statistically significant (Figure 6). Although it is not clear why whey protein may down-regulate estrogen synthesis, there may be a link to the activity of CYP 3A4 [30], or similar estrogen influencing biochemical pathways following whey consumption. The potential interaction of whey protein and estrogen synthesis is an area worthy of further study.

As previously discussed, isoflavones may have a significant effect on circulating levels of testosterone. To date 11 previous studies have evaluated the effects of isoflavones and soy on sex hormone levels [31,12,14,17,22,33,16,20], of which only two have shown a significant decline in testosterone [12,14]. Although the effects on total testosterone were minor, the relative response correlated to increasing isoflavone content. Gardner-Thorpe et al. gave 120 mg of isoflavones per day, a level comparable to the 138 mg/day supplementation for the SC group in the current study (Table 2). That the SC group was the only soy containing group showing a numerical reduction (not statistically significant) towards declining testosterone concentrations further suggests that a future study may want to evaluated if SC in a dose dependent manner affects serum testosterone levels. The biologically active and less commonly assessed free testosterone was not significantly reduced across all groups. Based on mean data, a trend towards a decline in the SC group was again evident (Figure 3). However, the lack of significance on circulating free testosterone levels corroborates previous studies on high protein soy isolates and soy-milk [15,16] but not for milk proteins [34,35] which may be as a direct result of differences in training methodology between studies.

The significant increase in the testosterone/estradiol ratio further contradicts any suggested relationship with declining androgenic activity following soy consumption (Figure 4). Both the biological activity and systemic clearance are influenced by the binding capacity of SHBG [36-38]. Since SHBG may alter the balance of both bound and unbound estrogens and androgens and has a greater binding to testosterone, the lack of effect on serum testosterone (free and total) should be reflected in shifts in SHBG concentrations. In agreement with previous studies on protein sources of phytoestrogens (i.e. soy), the current study has shown no significant change (Figure 5) [14,19,16] in testosterone or SHBG. The lack of significant changes in SHBG may be due to confounding dietary variables, physical activity, lifestyle factors [39-41], and bodyweight [42]. Factors such as diet not controlled in the present study cannot be discounted. Although dietary intake (fat and calories) was significantly different between groups, 24 h dietary recall has significant limitations with regard to precision; nutrient intake estimates can ranging from 4 to 400% when compared with observed intakes [43]. However, work comparing 24 hr versus 3 day dietary record shows comparable results as general indicators of dietary intake [44]. Even with the use of biomarkers dietary analysis can prove inaccurate [44]. As this was a free-living study without controlled diets 24 hr recall was deemed an appropriate indicator of estimated dietary intake patterns, however precise nutrient intake records maybe considered a limitation of the current study. Although a free living based nutritional study design is efficacious to external validity, the integration of 30-day or multiple 24 hour recall assessment could be employed to better detect dietary influences on training and hormonal adaptation.

The resistance training protocol utilized in the present study effectively increased the accretion of lean tissue irrespective of intervention. Increases in lean mass are consistent with the results of previous investigations [45,46]. The application of 12 weeks of resistance training was based on previous research describing the occurrence of significant muscle hypertrophy within this time frame [47,48] and that significant diet-related differences in muscle hypertrophy occur within this period [47,48]. The results of the present study confirm these earlier studies regarding adaptive responses to diet and training.

No consistent effects were evident in the fatigue and vigor scores, although the POMS-Vigor score increased significantly in the highest isoflavone containing group (SC). Although, previous work has shown improvements in mood with exercise [49] and mood in females following isoflavone consumption [50], this is the first study to show any effect on males.

## Conclusion

The current randomized and blinded intervention study in healthy men demonstrated that protein, irrespective of source (SI, SC, SW or WB), coupled with resistance training results in a significant enhancement of lean body mass. There was no significant decrease in serum androgenic hormones following supplementation with any protein intervention. The biological significance of the sex hormone changes within the current study resulting from lower estradiol, and increased testosterone/estradiol ratio in response to soy and whey supplementation is unknown at this time. The isoflavone content may affect the magnitude of hormonal change as may the duration of dietary exposure to supplemental isoflavones.

This is the first study to report low-level isoflavone content in commercial whey proteins, and future studies should take this into consideration when looking to designing an isoflavone free control study. Previous work has shown occurrence of isoflavones in the range of 1–30 ng/ml in bovine milk [51] suggesting that whey may not be an isoflavone free protein source. Although the sex hormone measurements were not statistically different in comparison to soy, factors such as sample size and duration of supplementation may have influenced outcome. Other factors such as equol responders and non-responders were not addressed in the current study. Because equol is know to influence the biochemical responses to isoflavone containing proteins further study is needed to asses its impact on lean mass changes [52]. Because both protein source and bioactive content may bring about specific biochemical non-hypertrophic responses to exercise; such as increased antioxidant capacity [25], future work should look to measure performance markers to detect additional benefits from dietary protein supplementation. In conclusion, it appears that both soy and whey supplementation in free living resistance training men results in lean body mass accretion without negatively affecting serum androgen levels.
